# Supplementary material for: What predicts legislative success of early care and education policies?: Applications of machine learning and Natural Language Processing in a cross-state early childhood policy analysis
Source: PLoS One. 2021 Feb 11;16(2):e0246730. doi: 10.1371/journal.pone.0246730 (PMC7877598; doi:10.1371/journal.pone.0246730)
Supplement: Attachment — Submitted filename: Response to Reviewers.pdf [file pone.0246730.s001.pdf]

December 1, 2020

Dear Editors:

Thank you for this opportunity to revise our manuscript for your review. We sincerely appreciate your feedback and comments in improving our manuscript. We continue to remain committed to publishing our paper in *PLOS ONE*. We provide our response (in Arial) under each corresponding point raised by the academic editor (#1-3) and reviewers (#5). Additionally, we included specific excerpts (indented, in Times New Roman font) that reflect the changes made in the revised manuscript.

1. Please ensure that your manuscript meets PLOS ONE's style requirements, including those for file naming. The PLOS ONE style templates can be found at https://journals.plos.org/plosone/s/file?id=wjVg/PLOSOne_formatting_sample_main_body.pdf and https://journals.plos.org/plosone/s/file?id=ba62/PLOSOne_formatting_sample_title_authors_affiliations.pdf

RESPONSE: We have carefully checked the links provided and compared to our manuscript in LaTeX. All figures have been converted to TIFF files.

---

2. Please update your submission to use the PLOS LaTeX template. The template and more information on our requirements for LaTeX submissions can be found at http://journals.plos.org/plosone/s/latex.

RESPONSE: We have used the PLOS LaTeX template and also have submitted the TEX file.

---

3. We note that Figure 3 in your submission contains map images which may be copyrighted. All PLOS content is published under the Creative Commons Attribution License (CC BY 4.0), which means that the manuscript, images, and Supporting Information files will be freely available online, and any third party is permitted to access, download, copy, distribute, and use these materials in any way, even commercially, with proper attribution. For these reasons, we cannot publish previously copyrighted maps or satellite images created using proprietary data, such as Google software (Google Maps, Street View, and Earth). For more information, see our copyright guidelines: http://journals.plos.org/plosone/s/licenses-and-copyright.

We require you to either (1) present written permission from the copyright holder to publish these figures specifically under the CC BY 4.0 license, or (2) remove the figures from your submission:

RESPONSE: The map image in Figure 3 of our manuscription is not copyrighted. We created this image by downloading a US Bureau of Census shape file, merging it with our data by state

and plotting the resulting quantities using the open source software R published and distributed under the GNU General Public License version 3 (The “GNU General Public License” version 3) and the open source visualization library ggplot published and distributed under the GNU General Public License version 2 (The “GNU General Public License” version 2). Consequently, the figure in question is no different than a histogram or any other visualization created and published in most journal articles. We therefore believe that the steps you have outlined in your feedback are unnecessary in this particular case. We updated the figure caption with source information as requested.

---

5. Review Comments to the Author

Reviewer #1a: Please illustrate further on the preprocessing of your text data.

RESPONSE: Thank you for your helpful feedback. We have added a new section “text preprocessing” between the descriptions of the data source and our analytic strategy as follows:

> **Text Preprocessing**
>
> Prior to feeding the text of the legislation into the LDA algorithm, we have taken several steps to extract the most helpful words from the text and remove redundant ones. Prior to using the text as input for the LDA algorithm[1], we excluded common “stop words” such as ‘the’ ‘and’ ‘or’ ‘because’ which could belong to any topic and as such do not contribute to the algorithm’s primary purpose of classification of text into topics. In general, we have removed all prepositions, conjunctions, interjections, and articles. Additionally, we have intervened to remove some grammatical features not relevant for topic classification such as declension and conjugations--in English this comes down to removing ‘s’ from plural form of noun and ‘s’ from third person singular of a verb. Hence, we removed such “stem words” that have the same root meaning (e.g., ‘children’ becomes ‘child’; ‘preschools’ becomes ‘preschool’). Thirdly, we removed proper names of states, cities, etc. These are a subset of proper nouns, although we have kept some as some proper nouns do have relevance in the Early Childhood Education space. For example, the Department of Health and Human Services is a proper noun with relevance to topic classification since certain matters in ECE are handled by this department while others are handled by the Department of Education (which we have also preserved). We also removed all the numbers, which we found irrelevant to topic classification. Some of these were references to other pieces of legislation, years, dates, etc.
>
> Finally, we fixed typos when we were able to find them and removed infrequent and obviously (based on our knowledge of ECE) irrelevant words such as ‘acupuncturist’ or ‘ammoniated’ in an effort to provide a better signal-to-noise ratio to the LDA algorithm to work with[2]. While removing words such ‘ammoniated’ is to some extent a

[1] List of stop words eliminated is available from authors upon request.
[2] We keep a list of such words so that researchers interested in replicating our results may do so reliably. The topics identified with those extra words were not fundamentally different but rather a bit more ‘noisy’

subjective decision, we do not claim that the topics we have discovered are 'true' or 'unique' in any sense. If one was to add 10 new pieces of legislation to the dataset, the topics discovered by LDA will be slightly altered. What we do claim is that the topics we identified through a combination of objective methods and subjective professional judgement have statistically significant relationship to the probability of passage of legislation and interact with other predictors of legislation success. In doing so, we demonstrate that standard predictors of legislation have a differential impact on the legislation's success depending on the topic of the legislation and this should be taken into account when considering strategies for promoting legislation related to the welfare of children and education of the nation.

Reviewer #1b: I believe the illustration on the document-word matrix can be expanded further. Readers may wonder how authors created the matrix. Please elaborate further on how authors assign the meaning of the topics. It's not clear from the manuscript.

RESPONSE:

Document-word matrix and how it was created

Thank you for your question. We had a bit of a hard time understanding this particular question. The phrase 'document-word matrix' doesn't appear anywhere in the manuscript and we have not really commented on it at all. Literal reading of your question would lead us to describe the document-word matrix, where the document is a piece of legislation and word is a word appearing in that legislation. This matrix would then have as many rows as there are pieces of legislation (3,203) and a number of columns equal to the number of words in all pieces of legislation combined. This would be a very sparse matrix as a minority of the total collection of words appears in any particular piece of legislation. This would be a frequency matrix where the numbers inside the matrix would represent how often a particular word appears in a particular document. We do not operate on this matrix ourselves but rather feed this matrix to the LDA algorithm, which operates on it. This matrix was created using the R software, whereby the original dataset also had 3,203 rows but the columns were more of the standard variety such as year legislation was deliberated, party of the sponsor, etc, variables described in Table 1 & 2 of the manuscript. One of those variables is legislation text -- the whole legislation text is located in a cell of this dataset. This column with legislative text is then broken apart into multiple columns, each containing a single word. In practice, because this is a very sparse matrix, the data is actually converted into a long form, where one column is the legislation ID and the other column is a word. This long dataset has duplicates, since words can appear multiple times in the same piece of legislation. This dataset is then tallied so that each word-document pair appears only once and an additional column representing frequencies is added. There is not much we have to say about this matrix (the wide version of it, which has a bit more intuitive if inefficient [in terms of computer memory use] structure) other than that the number of columns is partly

---

or 'blurry' in a sense that there was a bit of an overlap between different topics. The LDA algorithm works best with large datasets (Nay, 2017 used 70,000 bills compared to our 4,000-5,000), hence we felt it may be helpful to ease its way into our text data using these pre-processing steps.

related to the number of words we filtered from the text, which is the subject of the previous question on text preprocessing, where this step is described. The reason why we do not have much to say about it is the same reason why we are using LDA in the first place. It is clumsy, too large, and at the end of the day, we do not care about how words affect the passage of legislation. Word is too small of a unit of text to carry much in a way of semantic features. This is why the LDA essentially compresses this matrix into a document-topic matrix with fewer columns and more meaning. In this response, we will try to describe various elements of our process that might bear some relevance on your question as we try to guess the intent behind your question.

Assignment of meaning to topics

We addressed this comment by incorporating substantial changes in the following sections: literature review, RQ#1 analytic strategy, and RQ#1 results. Assigning the meaning of the topics was driven by both theoretical and practical knowledge of the early childhood field as well as empirical evidence. Prior to analysis, we articulated the existing body of research and foundational theories in early childhood policy literature that informed our decisions in assigning and validating the topics. Please refer to the literature review section--"*Predictors of Legislative Success of ECE Bills: Policy Content & Topics*." Specifically, our systematic review explored governance and finance of early childhood services as prominent topics in both U.S. and global early childhood policies. We also included explicit language to communicate how the initial literature review helped inform our labeling of the initial meta-topics: 'finance' and 'services':

> In this paper, we will be exploring the content of legislation through a combination of literature review and natural language processing. Our literature review work will identify conceptually meaningful topics that could potentially inform topic assignment and validation in interpreting the results produced by the natural language processing algorithm used in this paper. For example, governance and finance of early childhood services are prominent subjects in legislation and function as critical mechanisms for providing equitable access to high quality services for children and their families (Britto et al., 2014)...
>
> Given this extensive focus of the early childhood field on these issues, one would naturally expect the possibility of these topics occurring in the ECE legislation as well. Hence, when we later present the modeling process and employ our natural language processing algorithm, we will discuss in depth how the extant literature informed topic assignment and validation.

Rather than imposing these broadly known topics a priori, we were interested in exploring whether the prominent constructs or policy topics in ECE bills would uncover more nuanced and understudied yet meaningful topics when using LDA. LDA is particularly useful when learning the patterns of massive text data to identify emerging topics that are theoretically meaningful but potentially underexamined. We have made significant revisions and expanded the RQ1 analytic

strategy and RQ1 results sections (see below) to elaborate further on topic assignment and validation as can be seen below.

RQ1 Analytic Strategy:

We used Latent Dirichlet Allocation (LDA) (Blei et al., 2003) to analyze the text of 2,396 early care and education bills. As one of the text mining methods, LDA was appropriate for algorithmically identifying recurring patterns of language from unstructured policy text data (Grimmer & Stewart, 2013; Hindman, 2015). Machine learning approaches to data mining find patterns and regularities in the unstructured data or extract semantically meaningful information. LDA is a generative probabilistic three-level hierarchical Bayesian model for collections of discrete data. This modeling approach allowed analysis of patterns in legislative data to better understand and conceptualize its content. In particular, it identified groupings of words across various pieces of legislation that formed the 'topics,' which we refer to as policy priorities in the context of this study. The LDA model estimated the following: 1) latent constructs or topics of policy content across all bills, 2) the probability that a certain word was associated with a certain topic, and 3) the proportion of topics in any given piece of legislation . The algorithm produced an absolute probability that a certain word was associated with a certain topic (non-exclusive) and relative odds that a word belonged to one topic rather than another. The topic weights representing the proportion of topics in legislative texts were created as an aggregation of the frequencies of those individual words and their affiliation with topics. As an example, let us say that the algorithm determined that the word 'interaction' belongs to topic 1 with probability 70% and the word 'emotional' belongs to topic 1 with probability 30%. Then imagine that there is a piece of legislation consisting of only those two words. Then the algorithm will determine that this piece of legislation is 50% composed of topic 1. This example illustrates the nature of the aggregation of information from words to documents, trading technical accuracy for simplicity of presentation. In reality, there are many words and they belong to several topics with various probabilities and the aggregation is not a simple average but rather an integral over a region of probability space of a probability density function.

However, one of the limitations of LDA is that it arrives at topic classification through a frequency analysis outlined above while human approach to topics is based on semantic structures. There is no guarantee that the frequency analysis approach the LDA takes will derive theoretically meaningful constructs without human aid (Sun et al., 2019). To reconcile the machine and human approach, topic validation becomes essential for automated text analysis since ultimately the topics identified through LDA will be used in a narrative shared with fellow humans (the research community that will read this paper in our case). Additionally, the LDA is not designed to infer how many topics there

are. It required[3] us to specify the number of topics. Existing literature in early childhood policy broadly identified policy priorities relevant to 'governance,' 'finance,' of 'programs and services' (Kagan & Reid, 2008). These categories of early childhood policy priorities as represented and discussed in the literature are broadly defined and do not discuss more nuanced and specific policy priorities or strategies state legislatures might propose for systemic implementation. For RQ1, we aimed to discover early childhood policy priorities that conformed to the issues of 'governance', 'finance', and 'programs and services' as described above. More importantly, we improved prior studies that did not as thoroughly validate the topics derived from text analysis. While there is no universal standard to guide such decision making in assigning and validating topics, automatic content analysis may generate unreliable or invalid measures (Grimmer & Stewart, 2013; Wilkerson & Casas, 2017). Thus in the current study, we aimed to use both substantive and statistical evidence to conduct comprehensive validation for topics by 1) employing a series of models that specified 2 to 7 topics, 2) using fit statistics to aid our model selection as illustrated in Figure 4, and 3) finally, evaluating the content validity and coherence of policy topics that represented conceptually and practically meaningful themes.

Fully Automated Clustering (FAC) is a classification method used to simultaneously estimate the categories and then classify documents into those categories. Thus, we applied FAC to explore emerging topics and validated the output of a 6-topic classification model based on both substantive and statistical evidence. We then carefully read and coded the textual context for each set of the 75 high frequency words that were associated with each of the six topics to further establish meaning in guiding our analysis. In labeling each topic and inferring the commonality across these words, we conducted content validity checks of six topics containing high frequency words (Figure 5) that exclusively appeared in a given topic. In addition, we carefully assessed the intent and meaning of these words in the context of legislative text, examined consistent and varying examples of how these words were used in the legislation, and finally, how these words provided a conceptual basis and coherence for each of the topics. This subjective evaluation led to determining that the 6-topic model was optimal. In the results section, we illustrate the process and results for determining topics, establishing content validity and coherence of topics, and testing their predictive validity.

[3] One could see this as an advantage, since it allows us to summarize the text to a level of granularity or detail chosen by the researcher. If seen in this light, one could say that LDA 'allows' us to specify the number of topics. In our case, LDA allowed us to choose a topic classification that bears most relevance for legislation's success. One could see topic definition to be guided as the age of children served, in which case the topics would be: 'infant/toddlers', 'preschoolers', etc. Another classification could have been by activity: 'childcare' (emphasis on care), 'preschool' (emphasis on education), etc. LDA's guiding approach is frequency of words, which could be aligned with one such semantic classification but does not have to. We as researchers chose a classification provided by LDA that also had such a semantic element to it.

RQ1 Results:

We present RQ1 findings that resulted from first specifying the number of topics through a series of LDA models and using fit statistics to aid our model selection, and then evaluating the content validity and semantic coherence of policy topics. Drawing from policy text as big data, we aimed to identify the most prominent key ‘topics’ or policy priorities that constitute early childhood legislation that were considered during the most recent legislative sessions (2015-2018) for 50 states. We employed a series of models that specified 2 to 7 topics. First, a two-topic solution initially suggested two broadly defined topics we had identified in the literature review: ‘finance’ and ‘services.’ However, topic allocations were more precisely estimated and resulted in more specific policy priorities when using the six-topic solution. As displayed in Figure 4 and Table 3, a six-topic solution demonstrated the strongest fit theoretically and empirically based on BIC and AIC measures of model fit. However, the “best” model needs to capture the topics of interest to the researcher (Roberts et al., 2014; Wang, Paisley, & Blei, 2011). Model choice is typically based at least partially on subjective considerations similar to those in the qualitative research (Grimmer & Stewart, 2013). These six topics represented more nuanced and conceptually meaningful topics that belonged to the two cross-cutting ‘meta-topics’: ‘ECE finance’ and ‘ECE services.’

Having determined a final set of six policy topics, we further elaborate on the process of assigning the meaning of the topics and assessing their content validity. Topic validation becomes essential for automated text analysis since the LDA algorithm’s underlying frequency analysis may identify ‘word groupings’ as ‘topics’, however, these ‘word groupings’ may not strike a human observer as having a semantic structure. To ensure computer-generated topics captured topics that were theoretically and practically meaningful, two expert coders evaluated the content validity and semantic coherence of the six topics. Content validity denotes the extent to which the topics identify coherent and distinct sets of ECE policy priorities and measure conceptually sound constructs. Having expert coders who have relevant theoretical and practical knowledge was critical for assessing topics’ content validity (Sun et al, 2019). Figure 5 displays a unique set of 75 words with highest probabilities of appearing in each topic and that best distinguish the topics from one another. Using these high probability terms, we independently coded and labeled each of the six topics and assessed its coherence (how these words hold together as a construct) and the extent to which a given topic is meaningful and consistent with the literature. To further aid this process, we carefully read high probability terms in original legislative text, inferring its definition, meaning, and usage and their relation to the topic. Upon repeating this process for each model specification, we compared and discussed our qualitative coding decisions. We reached agreement on classifying most of the topics, and refined the label for just one topic, ‘Fiscal Governance,’ that integrated both the administration and financing of ECE services.

For ECE finance, we identified three specific topics -- 'revenues', 'expenditures', and 'fiscal governance'. Unique keywords with highest probabilities of appearing under **'revenues'** were *adjustment* (of schedule to facilitate department accounting or unit allocation), *agreement* (i.e., property tax or tobacco tax agreement), a*llocation, allowable tax credit, allowance* (for instructional classroom support) *business, calculation, corporation, credit, distribute, dollar, income, fiscal intermediary, properties, residence, sale, tax, taxable, taxpayers*. These words together suggested budgetary sources needed to finance early childhood care and educational services, particularly in schools and districts as seen in Figure 2. High frequency words for **'expenditures'** included *benefit, care, children, clients, disabled children, families, hospital, maltreatment, medical, medicaid, mental health, nurse* and *treatment*, suggesting what it would cost for various providers in delivering early childhood services including mental health and social welfare services for direct beneficiaries. Furthermore, several keywords, for example, indicated expenditures in terms of financing the early childhood workforce (i.e., structuring salaries and benefits). This process involves setting standards for teacher qualifications and other structural characteristics (e.g., class size, ratio, hours per week) of programs that affect cost. Since public expenditures on state preschool come from federal, state as well as local sources, states vary in their program schedule (half-day or a full-day) and leave the schedule up to local discretion (Barnett et al., 2009). Local authorities determine how thinly state funding is stretched regarding length of day and frequently, data are unavailable about how many children are served with each type of schedule. In managing state budget--both revenues and expenditures--the third finance-related topic emerged as **'fiscal governance'** dealing with *account, appropriation, balance, capital* (capital gain, capital expenditure, capital loss)*, commission, committee, contract, finance, item, local, money, officials, reimbursement, salaries, schedule, fiscal transfer* (by administrative bodies to accounts)*,* and *trust* (governing board of trustees as well as trust management). These words seemed to suggest the logistics of locating the money to pay for the programs at the legislative level and facilitating efficient distribution and use of resources. A commonly shared set of words that appeared across the three topics further confirmed ECE finance as the underlying theme--*amount, authorize, communities, cost, counties, department, fund, payment, provide,* and *public.*

For ECE services, three specific topics included governance and provision of early childhood services: 'prekindergarten (PreK),' 'child care,' and 'health and human services (HHS).' We found that three key topics--'prekindergarten' (preK), 'child care', and 'health and human services' (HHS)--addressed policy questions of *what* (types and quality of provision, program components, and dosage), *for whom* (targeted population, access and participation trends particularly among historically marginalized children and their families), *where* (delivery settings), as well as *by whom* (administration leaders and providers). Classification of these three topics closely reflect the complexity of early

childhood governance as well as the fragmentation and duplication of federal funding streams and state administration across early childhood programs and services. More specifically, unique words with highest probabilities of appearing for **'preK'** included *academic, achieve(ment), charter, college-ready, commission(er), district, enroll, grade, instruct, kindergarten, prekindergarten, preschool, pupil, school, student,* and *teacher*. In comparison, high frequency words under **'child care'** included *assist(ing), children* (rather than pupils or students under 'preK' topic), *counties, daycare, income, infant, subsidi(-es, -ze), and home visit*. These two topics, 'preK' and 'child care', mutually focused on promoting the development and early learning opportunities for eligible children through educational programming and services. Unlike the topics 'preK' and 'child care', **'HHS'** embodied a broader system of early childhood and social welfare services as suggested by words such as a*buse, adopt(ion), agency, child care, convict(-ed, -ion), court, criminal, daycare, foster care, health, home visit, neglect, personnel,* for example. The theory of change undergirding 'HHS' services posits that preparing children for success in school and life involve not only their cognitive development and academic enrichment but also their physical and mental health as well as social-emotional well-being and family support services. Types of services may include home visits, parent support or training, referral to social services, and health services. Additionally, examining a commonly shared set of words underlying these three topics further supported our classification of these topics having to do with ECE programs and services: Words such as *authorize, children, parents, program, provide, public, purpose, receive, require* and *services* further supported our conceptualization of these three topics as complementary yet distinct dimensions of providing public early childhood programs and services that are designed to promote children's learning and healthy development.

Reviewer #1c: Authors mentioned the multilevel structure of the data and used HGLM. I am just wondering whether LDA is OK for the multilevel data structure. Further, in general, is it ok to apply machine learning approaches such as LDA to multilevel data structure? Why?

RESPONSE: Thank you for raising this. We have addressed your question in the analytic strategy section for RQ2 as seen below.

> "If OLS was used for this type of data, the statistical significance of the regression results might be overstated, an issue which is not relevant in the LDA itself since statistical hypothesis testing is not being done for the topics themselves."

We have tried to avoid a lengthy treatment as this is an applied paper. Hence, we have opted to respond in detail below:

To preface the actual answer, let us review some fundamentals. Statistics is principally concerned with two overarching pursuits--*statistical inference* and *prediction*. Multilevel structure

has been identified as a potential issue in *statistical inference* as it is the part of statistics that is concerned with hypothesis testing, whereas prediction chiefly uses cross-validation (in effect, an out-of-sample prediction performance) to ascertain its suitability for the goal at hand. In statistical inference, we care about the 'beta' (quantifiers of the association between the outcome and independent variables) coefficients, about them being unbiased estimates of the population quantities in question, and about the standard error associated with the estimator being also unbiased. In particular, the issue with hierarchical modeling is that two observations from the same state (in this case) would not bring as much information as two observations from different states and that treating them as such might inflate the null hypothesis rejection rate in our analysis (find an effect too often). This is why in the inference section of our paper (RQ2 & RQ3), we have opted for HLM as we wanted to be conservative with our hypothesis testing and statistical inference. Another way of looking at this is that being conservative in inference means using a better model (with more realistic assumptions), while being conservative in prediction means using a 'worse' (a more simple) model (to avoid overfitting the training data and being less generalizable as a result).

The LDA model (RQ1) in our analysis serves as a *prediction* model, in effect synthesizing a variable later to be used as an independent variable in an inference model (RQ2 & RQ3). Let us play out a hypothetical scenario where we use an LDA in RQ1 and some other model B (could be a better version of LDA with incorporation of states or another model altogether) to identify topics. Let us assume that model B performs 'better' in a sense that the topics it discovers are clear-cut and contain fewer words that should really belong to another topic -- that it introduces a clean (as opposed to 'blurry') classification of topics in the legislation. Then for the inference purposes (RQ2 & RQ3) we would say that model B gives predictors that are less noisy. This will affect the inference in RQ2 & RQ3 via lower standard errors on the 'beta' coefficients associated with those topics. This, in turn, will make it more likely that those topics are statistically significant predictors of the legislation's success. To restate what we mentioned above, in inference, better modeling such HLM can sometimes nullify results identified with simpler models such as OLS. In prediction, better modeling does not nullify results.

A noisy predictor would make it harder to reject the null of no effect of topics on legislative success. In this sense, the reason why we are mindful of hierarchical structure in the data with RQ2 and RQ3 is because we are conservative, while the reason why this is not as much of an issue with RQ1 is that we only need a model 'good enough' to discover topics that in our disciplinary knowledge and understanding of the early childhood field appear a reasonable reflection of U.S. early childhood policies. Had we incorporated states in some sense as information into the LDA model, we could have increased the topic clarity and rejected the null of no association with even greater confidence in RQ2 & RQ3, but the conclusion would remain unaltered. With a better natural text processing model, the association between legislation's success and topics would be even stronger. Finally, by demonstrating the relevance of topics with even a bare-bones NLP model, we are offering the research community a lower bar on what can be achieved in a continued pursuit of NLP algorithms in application to early childhood policy analysis. Given the NLP algorithms are still an active area of research, our paper gives the ECE community a preview and a lower bar on the benefits that come with it.

A more detailed treatment of the issues discussed above can be found in:

- Hastie, T., Tibshirani, R., & Friedman, J. (2009). *The elements of statistical learning: data mining, inference, and prediction*. Springer Science & Business Media.
- Gelman, A., & Hill, J. (2006). *Data analysis using regression and multilevel/hierarchical models*. Cambridge university press.

Reviewer #2: This paper presents an original contribution to the field through an analytic approach implementing machine-learning to unlock the potential of legislative data to inform future policymaking in early childhood-care and education.

The rationale for the research question is clearly stated. The general approach and statistical analyses were described and carried out rigorously. The discussion and conclusion appropriately derive from the data presented.

Although "the use of big data in research on public policies has lagged behind other fields", it is important to highlight some recent studies which applied machine-learning to predict educational outcomes at large scale and/or educational data mining field (for example: Musso MF, Cascallar EC, Bostani N and Crawford M (2020) Identifying Reliable Predictors of Educational Outcomes Through Machine-Learning Predictive Modeling. Frontiers in Education. 5:104. doi: 10.3389/feduc.2020.00104, and Romero, C & Ventura, S. (2017) Educational Data Science in Massive Open Online Courses. Wiley Interdisciplinary Reviews: Data Mining and Knowledge Discovery · January 2017).

RESPONSE: We appreciate Reviewer #2 for sharing these relevant references and specifying where we might cite them. We agree that highlighting such emerging studies fairly acknowledges emerging efforts in the educational data mining field and further enriches our discussion of findings and future directions for research. We have incorporated your suggested sources along with others to highlight emerging applications of topic modeling in educational research both in the literature review and the discussion section of our revised manuscript (see revised excerpts below):

> “Topic Modeling (Blei et al., 2003) is a rapidly emerging area of research in educational data mining. Text analysis methods in education research have been used to 1) measure latent dispositions such as attitudes and beliefs of learners and instructors (U.S. Department of Education, 2012); 2) explore the underlying topics and topic evolution spanning the 50-year history of educational leadership research literature (Wang et al., 2017); 3) microclassroom processes such as MOOC interaction data (Romero & Ventura, 2017); 4) policy implementation and reform strategies (Sun et al., 2019); or 5) constructing artificial neural network models of multiple setting-level predictors of student’s language and math achievement outcomes (Musso et al., 2020). Despite the promising potential of applying topic modeling in a variety of fields in the social sciences, its scalable, algorithmic approach to large-scale text data has received little attention among early childhood and education policy scholars (Wang et al., 2017).”

> “While the use of big data broadly in research on public policies and publicly-funded programs has lagged behind other fields such as computer science and medicine (Coulton et al., 2015), LDA in particular has been utilized in understanding legislative policymaking (Blei et al., 2003; Nay 2017). Additionally, the application of computer-assisted analyses of large-scale text such as text reuse methods (Wilkerson et al., 2015), topic modeling (Blei et al., 2003) and classification methods are far sparse in education policy research (Sun et al., 2019). However, a burgeoning body of work has applied machine learning algorithms in educational research to better understand how teaching and learning can be enhanced for whom under what conditions (Elatia et al., 2016; U.S. Department of Education, 2012). Recent studies, for example, have built artificial neural network models that predict students’ language and math performance at large scale (Musso et al., 2020), introduced various methods in educational data science (EDS) for examining students’ massive open online courses (MOOCs) interactions (Romero & Ventura, 2017), or using LDA to analyze text data from thousands of school improvement reports to identify reform mechanisms that reduced student chronic absenteeism and improved achievement (Sun et al., 2019).”

While revising your submission, please upload your figure files to the Preflight Analysis and Conversion Engine (PACE) digital diagnostic tool, https://pacev2.apexcovantage.com/. PACE helps ensure that figures meet PLOS requirements. To use PACE, you must first register as a user. Registration is free. Then, login and navigate to the UPLOAD tab, where you will find detailed instructions on how to use the tool. If you encounter any issues or have any questions when using PACE, please email PLOS at figures@plos.org. Please note that Supporting Information files do not need this step.

RESPONSE: Thank you for this information. We have uploaded our figure files to the PACE digital diagnostic tool to ensure they meet PLOS requirements.
